# Learning from Bees: An Approach for Influence Maximization on Viral Campaigns

**DOI:** 10.1371/journal.pone.0168125

**Published:** 2016-12-19

**Authors:** C. Prem Sankar, Asharaf S., K. Satheesh Kumar

**Affiliations:** 1 Department of Futures Studies, University of Kerala, Kariavattom, Kerala, India - 695 581; 2 IIITM-K, Thiruvananthapuram, Kerala, India - 695 581; Semmelweis University, HUNGARY

## Abstract

Maximisation of influence propagation is a key ingredient to any viral marketing or socio-political campaigns. However, it is an NP-hard problem, and various approximate algorithms have been suggested to address the issue, though not largely successful. In this paper, we propose a bio-inspired approach to select the initial set of nodes which is significant in rapid convergence towards a sub-optimal solution in minimal runtime. The performance of the algorithm is evaluated using the re-tweet network of the hashtag *#KissofLove* on Twitter associated with the non-violent protest against the moral policing spread to many parts of India. Comparison with existing centrality based node ranking process the proposed method significant improvement on influence propagation. The proposed algorithm is one of the hardly few bio-inspired algorithms in network theory. We also report the results of the exploratory analysis of the network kiss of love campaign.

## Introduction

Social networking platforms, such as Facebook and Twitter, have been extensively used for socio-political movements apart from viral marketing campaigns in the recent past as an increasing number of people spend more time online. In this scenario, it has become a challenge for the campaigners to diffuse the information quickly across the network. The influence maximisation problem originally introduced in the context of viral marketing is NP-hard for obtaining an optimal subset of users who can maximise the information diffusion [1–3]. It has resulted in an abundant development of approximate algorithms to identify prominent actors or the so-called *super spreaders* [4–6]. Study of such collective intelligence of individual users in socio-political campaigns will expand our capability to judge the human social behaviour and explain the influence diffusion process. Campaigns starting with people, who are the most influential individuals in society (also called initiators, influencers, key players, opinion leaders *etc.*) would lead to the maximum possible diffusion in minimum time. Hence, it is crucial for any campaign to select a small set of influencing seed users who have a strong influence on others. In this work, we propose a bio-inspired approach to select nodes in the initial set which can lead to a more rapid information propagation process and to simulate the diffusion by identifying retweet process of Twitter campaigns with the waggle dance of a bee colony.

Wu *et al.* have studied the spread of information in social groups and identified information relevant to one person is more likely to be of interest to individuals in the same social circle than those outside of it [7]. Information can be quickly spread between individuals within the micro-blogs in the form of word-of-mouth communication. Jansen *et al.* have examined Twitter as a medium for electronic word-of-mouth advertising and discuss the implications for corporates using micro-blogging as part of their overall marketing strategy [8]. Twitter hashtag adoption is a unique form of folksonomy since the initial adopters of the hashtag can be viewed as innovators and they attract or influence another group of users, namely, imitators [9]. Meeyoung and Haddadi compared three measures of influence: in-degree, retweets, and mentions and hypothesised that number of followers might not be a good measure of influence [10]. Besides, influence is not gained spontaneously or accidentally, but through concerted effort such as limiting tweets to a single topic. Weng and Jianshu proposed a topic—sensitive homophile based Page-rank measure for identifying influential users in Twitter and their *TwitterRank algorithm* measures the influence taking both the topical similarity between users and the link structure into account [11]. Gonzalez-Bailon *et al.* identified four types of users—namely, influential, hidden influential, broadcasters and common users—which can help the understanding of how users behave in diffusion processes [12]. Ramasuri and Narahari have applied cooperative game theory concept *shapely value* for solving the influence maximisation problem [13].

Nature was a source of inspiration for developing alternate algorithms for solving many real-world optimisation problems [14]. Bio-inspired algorithms, a major class of such algorithms, are popular mainly due to their efficiency. Swarm intelligence (SI), a branch of artificial intelligence (AI), is concerned with the design of intelligent multi-agent systems by taking inspiration from the collective behaviour of social insect colonies and other animal societies interacting locally with one another and with their environment by applying the concept of decentralised control and self-organization [15].

One of the major highlights in swarm intelligence domain is the development of ant algorithms for discrete optimisation in 1999 [16]. In 2003 modelling based on a natural behaviour of social insects was used for transportation problems [17]. Rivero *et al.* applied a biologically-inspired modification of ant colony optimisation algorithm for path search in social networks [18]. They extended the standard ACO algorithm by equipping the ants with a sense to smell similar to ant’s natural pheromone-tracking capabilities. The extension adds further ability to follow the trail of food odour to the source. Bojic *et al.* have demonstrated recently how firefly synchronisation can be utilised for clustering of networks as well as data diffusion in machine networks [19]. Gao *et al.* proposed bio-inspired methodology, combining *physarum centrality* and *K-shell index*, to identify the influential node in a weighted network and compared its performance with other methods [20]. In the same year, an improved *Particle Swarm Optimisation (PSO)* algorithm was proposed by Zhang *et al.* to find the most influential users of Sina Weibo, the popular microblogging service in China [21]. Their algorithm utilises social interaction pattern to find an optimal solution. In Sina Weibo, users participate in network interaction by publishing tweets and retweets. The retweeting behaviour can be described as a variable of a user influence space which contains user experiences and surrounding network. Nikolaev *et al.* have recently introduced a metric, *viz.*, *engagement capacity*, as a measure of the users to engage peers, to characterise online forum user behaviour and analyse the reach maximisation of online social media platforms [22]. Achananuparp *et al.* have taken the number of retweets as the engagement of Twitter user [23].

Among many swarm intelligence algorithms, Artificial Bee Colony (ABC) is the one which has been most widely studied and applied to solve the real world problems. Bees algorithm, introduced in 2005 by Dervis [24], is an optimisation algorithm inspired by the natural foraging strategy of honey bees. Each candidate solution is considered as a food source (flower), and a population (colony) of agents (bees) is used to search the solution space. An artificial bee visits a flower (lands on a solution) and evaluates its profitability (fitness). The Artificial Bee Colony (ABC) algorithm introduced by Karaboga *et al.* in 2007 follows a new meta-heuristic approach inspired by the natural foraging behaviour of a honey bee to find the optimal food resources through a *waggle dance* [24–26]. A colony of honey bees constantly search the environment looking for new flower patches. Bees that found a highly profitable food source go to an area in the hive called the *dance floor*, and perform *waggle dance*. Through the waggle dance, a scout bee communicates the location of its discovery to other bees, which join in the exploration of the flower patch. Since the length of the dance is proportional to the scouts rating of the food source, more bees get recruited to harvest the best-rated flower patches. In 2009, Singh extended artificial bee colony algorithm for solving a constrained optimisation problem in minimum spanning tree [27]. In 2011, Pan *et al.* presented a discrete version of ABC for the lot-streaming flow shop scheduling problem [28]. Omkar *et al.* introduced a generic model based on the ABC for multi-objective design optimisation [29] in the same year. Akay and Karaboga introduced modified versions of ABC algorithm and applied them for efficiently solving real-parameter optimisation problems [30]. The modification is often related to the ratio of variance operator and the frequency of perturbation. Wu *et al.* presented an improved ABC algorithm to enhance the global search ability of basic ABC [31]. ABC algorithm was later upgraded for constrained optimisation problems [32]. Xu *et al.* described a chaotic ABC approach and applied to path planning of uninhabited combat air vehicle (UCAV) in various combat fields [33]. Zhang *et al.* modified ABC algorithm by changing the stages of *employed* and *onlooker* bees to promote the convergence rate [34]. Bio-inspired algorithms have many advantages such as the ability to provide multiple solutions, suitability of implementation in a parallel computing environment in addition to their capability of overcoming many shortcomings of traditional algorithms [35]. However, there are hardly few bio-inspired algorithms dealing with approximation problems in social network scenarios, the influence maximisation problem in particular.

In this paper, a new approach is proposed for simulation of influence propagation in viral campaigns through online micro-blogging platform Twitter for selecting a set of seed nodes which lead to maximum diffusion of influence in a particular context combining with a new node ranking procedure. Our approach is inspired by *waggle dance*, a communication process of honey bees, and utilises the global-local search capacity of the ABC algorithm to solve the influence maximisation problem. The effectiveness of the method is tested employing a retweet network of the hashtag *#KissofLove* associated with one of the first socio-political movements in India which used Twitter very effectively.

## Proposed Method

It is extremely tough to identify the influential users during a collective action in micro-blogs without considering the features of tweets and user profiles. The proposed method focuses on approximating the optimal solution of influence maximisation problem using principles of swarm intelligence. The information available for each user is based on its activities and the knowledge of other individuals in the neighbourhood. Influence maximisation problem can be addressed by analysing the user, message, network and temporal features. This work combines all these features by analysing the tweet corpus and introduces a node ranking process by applying the concept of social interactions to find the optimal solution.

The ABC algorithm mimics the collective foraging behaviour of honey bees on searching food source. The central component of an ABC algorithm is combination global-local search feature and waggle dance process. Through information exchanging and learning mechanism, the whole colony would always find relatively prominent food source. The ABC algorithm can be suitably adapted to gain useful knowledge from Twitter networks. Key personalities in social media who perform a *waggle dance*, by retweeting a particular tweet to attract their followers. We consider retweet of a particular tweet by a key user as a waggle dance. These key personalities may perform a *waggle dance* to attract their followers to a particular campaign. A user retweets a particular tweet to express his or her interests, beliefs, thoughts and concerns. So the social influence of Twitter profile and the favourability of tweet motivates one to retweet the tweets that help to represent their common interests. Such messages will be propagated within the social network through the connections between people, creating a good campaign that is highly personalised and engaging the interests of such users can almost be guaranteed to go viral.

The selection of the initial set of nodes from the network to start with the diffusion process play a crucial role in the influence maximisation problem. In general, the initial set is selected randomly. However, in this work we propose a bio-inspired approach for the selection of the initial set of *k* nodes which is significant in rapid convergence towards a sub-optimal solution in minimal runtime. Our approach, rather than selecting random k-seed nodes, introduces a node ranking process to rank the nodes by combining user profile features and message activities in the context. The proposed approach is divided into two parts: in the first part each node is ranked according to social reputation value and in second part top *k* nodes are selected as the initial *k*-*node set*. The diffusion process simulated with ABC algorithm detailed below.

### Node Ranking Process

The reputation of social profiles is the engine which powers any social media campaign. Profile features are used to identify the initial influence of each user and the influence value is updated based on tweet and retweet relationships. Finally, the reputation of each user is calculated based on total social activities in particular context. This social reputation rank is used to select the initial k-seed set. It is also used to select nodes for further exploration in decreasing order of their reputation rank in the network as detailed in Algorithm 1.

**Algorithm 1:** Procedure for finding reputation rank of nodes

 **Data:** Tweet corpus with a particular hashtag

 **Result:** Reputation rank of each node

1 List all nodes in constructed digraph;

2: InitializeInfluenceValue();     ▹ for calculating profile influence value;

3: **while** *Tweet corpus is non empty* **do**

4:  **if** *Message is a retweet* **then**

5:   UpdateReTweet();

6:  **else**

7:   UpdateTweet();

8:  **end**

9:  UpdateInfluenceValue();

10: **end**

11: UpdateReputationRank();

The InitializeInfluenceValue() function is used to initialize the profile influence value of all nodes of the constructed digraph. Initially, each node has a slight influence value (Inf_0_) based on profile parameters such as the number of followers and number of users followed, shortly, *following*. Every user should have a sufficient number of followers to propagate his influence through tweets. However, more followers do not necessarily mean more influence. The initial influence (Inf_0_), of each user, is calculated according to

$$\begin{array}{c} {\mathrm{Inf}}_{0}=\frac{\text{Number}\;\text{of}\;\text{Followers}}{\text{Number}\;\text{of}\;\text{Following}} \end{array}$$ (1)

and is assigned as the reputation of individual profiles. The celebrities and newsgroups profiles may have a huge number of followers, but they may follow very less number of people and hence Inf_0_ can take large values in the initial stage. Cha *et al.* have demonstrated that having a large number of followers does not contribute much to the influence of a user in the Twitter world whereas the number of users who actively retweet counts more [10]. Therefore, to reduce the significance of the number of followers in Inf_0_, we introduce a normalisation process with parameters, namely, *Critical Value (CV)* and *Threshold Value (TV)*. The values of these parameters can be fixed based on the variation of Inf_0_. The new normalised influence value is calculated as follows

$$\begin{array}{c} {\mathrm{Inf}}_{0}^{\prime }=\frac{{\mathrm{Inf}}_{0}}{CV} \end{array}$$ (2)

If ${\mathrm{Inf}}_{0}^{\prime }$ value is greater than the *threshold value (TV)* then it is normalized again with *TV* using

$$\begin{array}{c} {\mathrm{Inf}}_{0}^{\prime \prime }=TV+\frac{{\mathrm{Inf}}_{0}^{\prime }}{{\text{AveInf}}_{0}^{\prime }} \end{array}$$ (3)

where ${\text{AveInf}}_{0}^{\prime }$ is the average of ${\text{Inf}}_{0}^{\prime }$ over all nodes. After calculating the initial influence, each message in the dataset is explored to update the influence value by using UpdateTweet() and UpdateRetweet() functions which updates the tweet and retweet count respectively of the corresponding author node. The retweets received indicates the influence of a user. People retweet what they are interested. The more a tweet is retweeted, the more influence that tweet gets. Therefore, users who have more forwarded tweets can be considered influential users in normal situations.

If the user A retweets a tweet of user B, then the influence of user B is incremented:

$$\begin{array}{c} \mathrm{Inf}{\left(B\right)}_{t+1}=\mathrm{Inf}{\left(B\right)}_{t}+\mathrm{Inf}{\left(A\right)}_{0}. \end{array}$$ (4)

This process is repeated until every message is processed. The final reputation rank of a user X in the social context is calculated iteratively:

$$\begin{array}{ccc} {\mathrm{Rep}}_{\text{t}}(X) & = & {\mathrm{Inf}}_{\text{t}}(X)-{\mathrm{Inf}}_{0}(X)\\ & & +\frac{\text{Number}\;\text{of}\;\text{Tweets}(X)}{\text{Total}\;\text{number}\;\text{of}\;\text{Tweets}}\\ & & +\frac{\text{Number}\;\text{of}\;\text{re-tweets}\;\text{received}}{\left(\text{Number}\;\text{of}\;\text{re-tweets}\;\text{send}+1\right)} \end{array}$$ (5)

### ABC Algorithm for influence Maximization

The starting point of any viral campaign on Twitter is the process of adopting a new hashtag. The small set of users recommend a particular hashtag in their tweets, their followers adopt it and retweet messages with the adopted hashtag, and it eventually becomes a widely recognised hashtag depending on the number of users adopting it.

**Algorithm 2:** Proposed algorithm for influence maximization in Twitter campaigns.

 **Data:** Retweet network with reputation rank in each node

 **Result:** K-Seed Nodes in hashtag context

1: Initialize *E* ⊂ *V* of employer bees with top k rank nodes;

2: Evaluate fitness value of each employer bee in *E*;

3: Initialize scout bees in *S* with the nearest neighbour nodes of employer bees in *E*;

4: Initialize onlooker bees in *O*;

5: **while** *termination condition is not met* **do**

6:  **for** *each scout bees* *i* *in* *S*     ▹ *starting with highest Rank*;

7:  **do**

8:   Calculate local fitness value of *i*;

9:   **if** *local fitness value of* *i* > *local fitness value of any* *j* ∈ *E* **then**

10:    update the status of *i* as employer bee;

11:    update the status of *j* as onlooker bee;

12:    Update the onlooker bee status;

13:   **end**

14:  **end**

15:  Update scout bee;

16: **end**

17: Calculate global fitness value;

18: Return the set of *k* employer bees;

A retweet network *G* = (*V*, *E*), shows tweet—retweet interactions associated with a particular hashtag within a group of individuals. It plays a fundamental role as a medium for the spread of information or influence among its members where *V* denotes the set of individuals (or nodes) and E denotes the set of edges. We consider a directed edge (*u*, *v*) ∈ *E* for any two nodes *u*, *v* ∈ *V* if *v* has retweeted any tweet of node *u*. In a typical influence maximisation scenario, one is interested to find *k* influencers to start with so that diffusion of influence is maximised. In the case of twitter campaigns the ultimate goal is to find the most engaging set of users from existing context who can be the initial set of adopters who lead to the most number of adoptions in similar socio-political campaigns in future. The analysis done by Cha *et al.* [10] have demonstrated that the most influential users can hold significant influence over a variety of themes. Here the problem is to choose a small set of influencing seed users who can get maximum influence on others. In many situations of collective action, only a few individuals among a group, for example, in the case of forage or travel, who have complete information, such as knowledge about the location of a food source, or of a migration route. Targeting these smaller proportion of informed individuals can achieve maximum diffusion of information within the entire network. The information diffusion happens through the social interactions that take place locally among the nodes that have direct connections.

We identify the analogy between individuals interacting on Twitter and bees perform waggle dance in a bee colony. The dynamic personalities in social media attract their followers to a particular campaign. We consider tweet—retweet activities of influencing user as a waggle dance which motivates us to adapt ABC algorithm to explore the retweet network to find the key influential nodes based on waggle dance. Each node in the given social context is considered as *flower patch*. The *employer bees,* who are used to locate influential opinion leaders in the network, are initially assigned to top *k* nodes from the node ranking process explained earlier. The *scout bees* are used to explore the nearest neighbour nodes of employer bees for better solutions. The *onlooker bees* indicate the followers (influenced) of influential opinion leaders. During the diffusion process, they are assigned with the status *influenced*. In each iteration the *local fitness value* is calculated by the maximum number of unique nodes that are influenced by a single node. The *global fitness value* is maximum unique influenced nodes by a group of *k* nodes.

Input to Algorithm 2 is the retweet network *G* = (*V*, *E*) with node reputation rank value *r*_*i*_ of each node *i* and a positive integer *k* and the output is a subset *A* ⊆ *V* with |*A*| ≤ *k* such that influence *A* is maximum. The proposed algorithm accept this input and initialize subset *E* ⊂ *V* of *employer bees* with nodes with top *k* ranks as the initial solution. Evaluate the local fitness value of each bee in *E* by counting the nodes that can be reached within *k*′ steps and update the onlooker bee status of these nodes to be *influenced.* Store these influenced nodes in set *O* ⊂ *V* of *onlooker bees.* In this process, if a node is already influenced by another employer bee then it is avoided. The set *S* ⊂ *V* of *scout bees* is initialized with the nearest neighbour nodes of initial employer bees. Now we conduct the local search for optimal solutions to the problem by searching the neighbouring nodes in each iteration. An iterative process then starts, with selecting a scout bee with the highest reputation rank from *S*. If the local fitness value of a scout bee is greater than that of an employer bee then the employer bee is replaced with the respective scout bee. The replacement process updates the onlooker and scout bee status of the bees. In next step we insert the nodes with distance two from an employer bee is inserted into current scout bee list and repeat above steps until every node is influenced or the number of scout bees becomes null. The iterative process of the fitness evaluation ends when some termination condition is met, such as exceeding the execution time limit or a certain ratio of the nodes being influenced. The result, which is the best *k* node set for influence maximisation, is then returned. The final set of employer bees is identified as the set of *k*–influential nodes in the network which can maximise diffusion in the given context.

## Results and Discussion

In this section, we report the results of the experiments with the proposed algorithm on twitter data associated with a hashtag *#KissofLove*. *Kiss of Love* protest is a non-violent protest against moral policing which started in Kerala and later spread to other parts of India. The movement began when a Facebook page named Kiss of love asked the youths across Kerala to assemble and kiss publicly as a protest against moral policing on November 2, 2014, at Kochi. The movement received widespread support in social media. After the initial protest in Kochi, similar protests were organised in the other main cities across the country. The protest was very popular on social networking sites and news media. Supporters of the campaign have been posting pictures of them kissing each other on social networking sites. Here we collected all tweets on online micro-blog Twitter under the hashtag #kissoflove and analysed to identify the most influential users.

**Fig 1:**
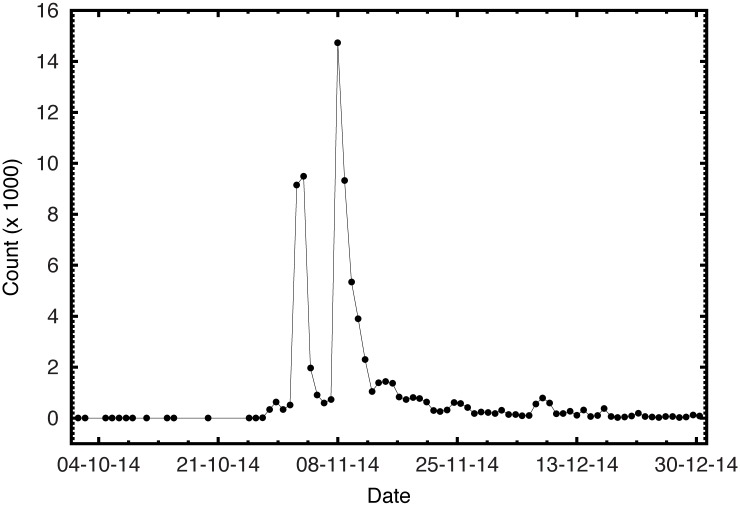
Growth of tweets in #KissofLove hashtag.

**Fig 2:**
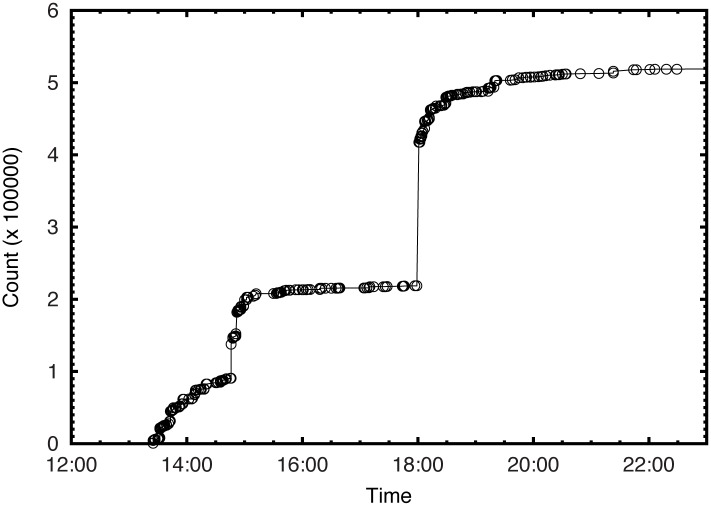
The reach of a typical tweet on 2 November 2014. The re-tweeet at 18:01 hours by an influential user with 198871 followers causes a sudden jump in the reach.

We collected 78,735 tweets from 52,570 users from Twitter with #kissoflove using Socioviz tool dated between October 1, 2014 and December 31, 2014 (*c. f.* Table 1) complied with the terms of service. The plot of the number of tweets per day in Fig 1 shows two breakouts. The first breakout happened during the first protest in Marine Drive, Kochi on 2 November 2014 with 9194 tweets and the second breakout occurred during the protest in Delhi on November 8 with 14371 tweets. Fig 2 shows a typical example of the diffusion of a single tweet in short span of time demonstrating the influencing role of re-tweets by the opinion leaders. The original tweet was retweeted by 350 times. However, a retweet by the user with 198871 followers at 18:01 hours causes a huge jump showing the impact of an influential user in the diffusion process.

**Table 1:** The details of the retweet network of *#KissofLove* protest.

Network	Nodes	Edges
Complete network	22738	36082
Giant component	16855	35357

A retweet network was constructed as a directed graph containing 22738 nodes representing all the active profiles and 36082 edges representing the re-tweeting relationships between these nodes. The network has a giant component with 16855 nodes and 35357 edges and 5168 islands. The largest among the islands has only 67 nodes. We considered the giant component for further analysis. As a first step, we computed the structural properties of giant component and is given in Table 2

**Table 2:** Structural properties of giant component.

Structural Property	Value
Average Degree	2.01
Network Diameter	19
Avg Clustering Coefficent	0.029
Average Path Length	6.382

**Fig 3:**
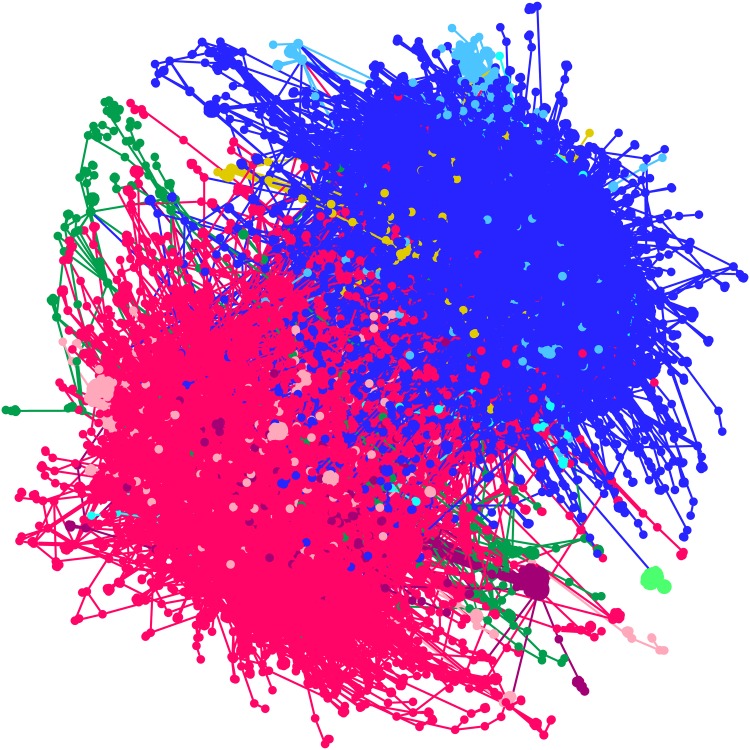
Clusters of re-tweet network. The two major clusters in blue and red colours represent supporters and opposers respectively.

The giant component was further subjected to cluster analysis [36, 37] with resolution parameter 2.0. A cluster in a network is a group of similar nodes. Here the objective is to understand how opinion on this movement is divided. The main clusters are shown in Fig 3. Content analysis of the tweets was carried out to understand the general characteristics of each cluster. The cluster of nodes in blue colour represents supporters (43.02%) and the cluster in red colour are opponents (55.99%), and this composition is interesting in a sociological perspective. The remaining clusters are not significant.

As the first step in the implementation of proposed algorithm, a set of top 10 nodes (identified by ID) of reputation value (Rep_t_), without normalisation procedure given in Eqs (2) and (3), is selected and is given in Table 3. It is evident from the table that media houses and celebrities have got high influence because of their large number of followers whereas they are following less number of people. As noted earlier the number of follower does not contribute much to influence diffusion in terms of retweets. As these users seldom retweet they were avoided from initial set by the normalisation procedure discussed previously and set both the *critical value (CV) and the threshold value (TV)* to be 10 for the implementation of the algorithm. The normalisation process is introduced to provide priority to individual users other than media houses. Top 10 users according to final reputation rank are shown in Table 4. It may be noted that some media houses still retains its position regarding reputation value showing their potential to contribute to the diffusion process. The reputation value indicates the worthiness of a specific user within a specific context or campaign. With the selected seed set of 10 nodes, the influence propagation is simulated according to Algorithm 2. Local search or exploration process analyses the neighbour nodes of a seed node and if they have higher fitness value than that of the seed nodes then update the existing list of seed nodes with new nodes. This procedure simplifies the computation by selecting initial nodes by the node ranking process to obtain a feasible estimate. The proposed procedure combines the fitness value obtained from local and global exploration to find the optimal solutions. This procedure is repeated until every node is explored. Here the algorithm returns final ranks of the users base on Rep_t_. The list of nodes with top ten ranks as given Table 5.

**Table 3:** Top ten profiles with reputation value (Rep_t_) without normalisation.

Rank	ID	Followers	Following	Tweets	Retweets	Retweets Received	Rep_*t*_	Profile Type
1	18716	144481	1	6	0	265	246.8541	Cricket Celebrity
2	3380	8395159	61	2	0	69	235.6157	News
3	13235	108723	0	1	0	11	188.2344	News
4	9325	227290	4	1	0	21	103.1516	Fashion Celebrity
5	1100	429436	12	2	0	14	68.6661	News
6	20506	1750558	52	2	0	24	65.1878	News
7	6118	526340	28	0	1	5	40.8162	News
8	13226	11691	0	2	0	4	29.1656	News
9	21054	10665	0	16	0	51	27.4836	Social blogger
10	4015	10252	0	1	0	8	26.8066	No details

**Table 4:** Initial top ten users identified by node ranking process based on Rep_t_ as per Algorithm 1 after normalisation.

Rank	ID	Initial Reputation (Inf_0_)	Tweets	Retweets	Re-tweets received	Rep_*t*_	Profile Type
1	12595	10.07094	40	80	840	3690.502	Social Blogger
2	22245	5.1356	21	110	1188	2712.612	Social Blogger
3	11626	10.70384	12	0	792	1448.124	News
4	10790	10.18415	65	0	233	1361.254	Social Blogger
5	19973	5.8342	18	0	496	1302.437	Journalist
6	6654	10.03122	15	66	332	1210.094	No details
7	16324	5.833	19	2	825	1128.391	Social Blogger
8	3379	10.12924	8	2	367	1013.984	News
9	2907	10.01723	16	15	255	850.2413	Social Blogger
10	11322	1.3535	32	37	342	817.9727	Social Blogger

**Table 5:** Final top ten users identified by Algorithm 2 with maximum influence in the network.

Rank	ID	Initial Reputation (Inf_0_)	Tweets	Retweets	Re-tweets received	Rep_*t*_	Profile Type
1	12595	10.07094	40	80	840	3690.502	Social Blogger
2	22245	5.1356	21	110	1188	2712.612	Social Blogger
3	15428	13.483	18	2	345	264.12	Social Blogger
4	21054	27.48361	38	10	322	495.482	RTI Activist
5	19973	5.8342	18	0	496	1302.437	Journalist
6	2907	10.01723	16	15	255	850.2413	Social Blogger
7	2	15.44	12	8	225	594.391	Social Blogger
8	9325	103.12	48	4	123	413.984	Social Blogger
9	11322	1.3535	32	37	342	817.9727	Social Blogger
10	6654	10.03122	15	66	332	1210.094	No details

To check the effectiveness of the proposed node ranking procedure, we have compared the selection of initial seed nodes based on our approach with standard ranking approaches for selecting the k-seed nodes based on different measures. We constructed the initial sets based on ten ranking measures, *viz.*, centralities such as degree, betweenness, closeness, activities such as tweeting and retweeting, and reputations such as Rep_t_. Starting with each initial seed node set, information diffusion was simulated using the giant component and computed the number of nodes influenced up to several iterations. The reach of influence with various initial sets of seed nodes is given in Table 6 in which the numbers shown in the cells are the number of nodes reached (informed) up to the iteration level starting with the set of initial nodes selected by corresponding ranking measure. The advantage of seed node selection based on Rep_t_, over all other measures, is clearly evident from the first iteration which is expected to improve the diffusion process through this initial set of adopters. After the first iteration, the diffusion could reach (the number of nodes informed) to 3683 nodes by initial seed node set selected according to Rep_t_ which is higher than that of the initial set selected by all other methods. It may be noted that values in the second row, obtained by initial set selected from taking the output of Algorithm 1, is closer to the values in the first row obtained by the initial set selected from the output of Algorithm 2. This shows that the seed nodes selected by the node ranking procedure introduced in this paper, considering social interaction in terms of tweets and retweets, is much closer to the optimum solution. The degree centrality does not reflect the global characteristics of a network whereas betweenness and closeness centralities do not consider the location of nodes [20]. Results of our simulations given in Table 6 show that out-degree and degree centralities perform comparatively better than betweenness and closeness centralities though they are inferior to Rep_t_. The results also show that the number of tweets or retweets alone of does not add value to a user profile for the diffusion process compared to other measures. The proposed procedure based on Rep_t_ has got the highest influence function value in the 7th iteration indicating a faster and wider diffusion of influence and hence more nodes may be influenced in subsequent iterations. It is evident from the table that the proposed reputation-based ranking method is superior for the rate of diffusion compared to other centrality measures based ranking. The influential nodes identified according to the bio-inspired approach introduced in this paper can be useful in future socio-political Twitter campaigns as Cha *et al.* [10] have observed that most influential users can hold significant influence over a variety of topics.

**Table 6:** Comparison of the rate of diffusion starting with seed nodes selected by different ranking methods. The numbers under each iteration show the number of nodes reached with respective ranking measure used for the selection of initial set.

Sl No	Ranking measure for seed node selection of initial set	Iteration number								
		1	2	3	4	5	6	7	8	9
1	Rep_t_(as per Table 5)	3683	6758	8537	9429	9929	9982	10017	10018	10018
2	Rep_t_(as per Table 4)	3503	6581	8623	9645	9898	9936	9970	9971	9971
3	Out-degree centrality	3246	6823	8729	9613	9815	9855	9877	9890	9890
4	Degree centrality	3497	6360	8438	9315	9784	9844	9865	9866	9866
5	${\mathrm{Inf}}_{0}^{\prime \prime }$	596	1561	3703	6195	7572	8301	8742	8778	8793
6	No of tweets	998	1340	4471	6749	7709	8153	8361	8417	8425
7	Betweeness centrality	1617	4140	6537	7738	8275	8349	8381	8389	8403
8	No of retweets	1220	3637	6294	7630	8231	8332	8380	8389	8403
9	In-degree centrality	786	2735	5701	7433	8182	8323	8354	8382	8389
10	Closness centrality	25	40	45	50	54	73	95	396	1157

## Conclusion

The quick diffusion of socio-political campaigns through online micro-blogging platforms such as Twitter is extremely dependent on identification seed nodes. In this paper, a new node ranking method is proposed and comparison, with indices popularly used for the same, indicates its advantage. The complexity of the exact influence maximisation problem has attracted many approximation algorithms in the literature of which bio-inspired algorithm are hardly few. We propose a bio-inspired algorithm identifying the retweet process with waggle dance of the bee colony. The performance is assessed with the retweet network formed by the #KissofLove campaign on tweeter. Results of the experiment show that the algorithm combined with the proposed node ranking method can effectively identify the opinion leaders online Twitter campaign. Besides, we also report results of the exploratory analysis the retweet network of the #KissofLove protest.
